# Comparative M-protein analysis of *Streptococcus pyogenes* from pharyngitis and skin infections in New Zealand: Implications for vaccine development

**DOI:** 10.1186/s12879-016-1891-6

**Published:** 2016-10-12

**Authors:** Deborah A Williamson, Pierre R Smeesters, Andrew C Steer, Julie Morgan, Mark Davies, Philip Carter, Arlo Upton, Stephen Y.C. Tong, John Fraser, Nicole J Moreland

**Affiliations:** 1 Institute of Environmental Science and Research, Wellington, New Zealand; 2 Microbiological Diagnostic Unit−Public Health Laboratory, Department of Microbiology and Immunology, The Peter Doherty Institute for Infection and Immunity, The University of Melbourne, Melbourne, Australia; 3 Murdoch Childrens Research Institute, Melbourne, Australia; 4 Labtests, Auckland, New Zealand; 5 Menzies School of Health Research, Darwin, Australia; 6 School of Medical Sciences and Maurice Wilkins Centre, University of Auckland, Auckland, New Zealand

## Abstract

**Background:**

Acute rheumatic fever (ARF) and rheumatic heart disease (RHD) are responsible for a significant disease burden amongst Māori and Pacific populations in New Zealand (NZ). However, contemporary data are lacking regarding circulating group A Streptococcal (GAS) strains in NZ. Such information is important in guiding vaccine development.

**Methods:**

GAS isolates from April to June 2015 were recovered from skin and pharyngeal samples from children living in areas of high social deprivation in Auckland, NZ, a significant proportion of which are Māori or Pacific. These children are among the highest risk group for developing ARF. Isolates were compared to concurrently collected pharyngeal isolates from Dunedin, NZ, where both the proportion of Māori and Pacific children and risk of developing ARF is low. *Emm* typing, *emm* cluster typing and theoretical coverage of the 30-valent vaccine candidate were undertaken as previously described.

**Results:**

A high diversity of *emm* types and a high proportion of *emm-*pattern D and cluster D4 isolates were detected amongst both skin and pharyngeal isolates in children at high risk of ARF. Pharyngeal isolates from children at low risk of ARF within the same country were significantly less diverse, less likely to be *emm* pattern D, and more likely to be theoretically covered by the 30-valent M protein vaccine.

**Conclusions:**

The high proportion of *emm* pattern D GAS strains amongst skin and pharyngeal isolates from children at high risk of ARF raises further questions about the role of skin infection in ARF pathogenesis. *Emm* types and *emm* clusters differed considerably between ARF endemic and non-endemic settings, even within the same country. This difference should be taken into account for vaccine development.

**Electronic supplementary material:**

The online version of this article (doi:10.1186/s12879-016-1891-6) contains supplementary material, which is available to authorized users.

## Background

Group A *Streptococcus* (GAS) is a major human pathogen, and infections caused by GAS are responsible for considerable global morbidity and mortality [1]. The spectrum of infections caused by GAS ranges from non-invasive infections such as impetigo and pharyngitis, through to invasive infections such as necrotizing fasciitis and toxic shock syndrome. In addition, acute rheumatic fever (ARF) and its sequela, rheumatic heart disease (RHD) continue to represent a considerable disease burden in many settings. This includes New Zealand, where the incidence of ARF in Māori and Pacific children is amongst the highest in the developed world [2].

At present, molecular typing of GAS is performed by sequence analysis of the 5’ hypervariable region of the *emm* gene that encodes the M-protein [3]. Recently, an *emm* cluster-based typing system was proposed, which classifies existing *emm* types into *emm* clusters based on genetic relatedness of *emm* protein sequences and functional activity of the M-protein [4]. This cluster system is predictive of *emm* pattern type, which, based on differential genotypic features and epidemiological associations, is a proposed marker for GAS tissue tropism [4]. In general, pattern A-C strains are associated with GAS pharyngitis, pattern D strains with skin infections (particularly impetigo), and pattern E strains with both oropharyngeal and skin infections [5].

In response to rising rates of ARF in New Zealand, the Rheumatic Fever Prevention Programme (RFPP) was set up by the New Zealand Ministry of Health. The RFPP is a multidisciplinary strategy aimed at reducing the incidence of ARF by two-thirds, to 1.4 cases per 100,000 population by June 2017 [6]. A large part of this strategy is focused on primary prevention of ARF, through the timely diagnosis and treatment of GAS pharyngitis. One of the models of care within this programme is the provision of school-based sore throat management clinics, with these services targeted at children at highest risk of ARF [6]. In New Zealand the distribution of ARF is unequal and those with the highest risk are Maori and Pacific and live in areas of low socio economic areas in the North Island [2]. The Counties Manukau region (South Auckland, North Island) has the highest incidence of rheumatic fever and the highest number of school-based sore throat clinics.

Recent epidemiological analyses have demonstrated that approximately half of ARF-associated GAS strains in New Zealand fall into pattern D–i.e. strains classically associated with skin infections [7]. Despite this association, and the high incidence of skin infections in New Zealand children [8], there have been few studies in New Zealand describing the circulating GAS *emm* types associated with superficial skin infections, and no contemporary studies comparing skin and pharyngeal isolates from high-risk children. In addition, it is not known whether there is any difference in GAS *emm* types circulating amongst children at high risk of ARF, compared to children with minimal risk.

The Coalition to Accelerate New Vaccines Against Streptococcus (CANVAS) is an Australian and New Zealand GAS vaccine development programme that aims to identify suitable GAS vaccine candidates, with the long-term aim of preventing GAS-related disease, particularly ARF/RHD and invasive disease [9]. At present, the most clinically advanced GAS vaccine candidates are those that target the N-terminal region of the M-protein, such as an experimental 30-valent M-protein vaccine [10, 11]. One of the early aims of CANVAS is to define the global epidemiology of circulating GAS strains, in order to better inform vaccine development and putative vaccine coverage.

Accordingly, the aims of this study were to: (i) describe the GAS *emm* types, *emm* clusters and patterns from children at high risk of ARF with presumptive GAS pharyngitis or skin disease in a New Zealand setting, (ii) compare the *emm* types of GAS strains from children at high risk of ARF with those at low risk, and (iii) determine the theoretical coverage of the 30-valent M-protein vaccine candidate against contemporary GAS strains associated with pharyngitis or skin disease.

## Methods

### Setting and population

Auckland is the largest city in New Zealand, with an estimated population of 1.47 million. The population is ethnically diverse, consisting approximately of the following major ethnicities in 2013: 57 % European/Other, 21 % Asian, 12 % Pacific Peoples and 10 % Māori [12]. Labtests Auckland (LTA) is the sole community diagnostic microbiology provider for the entire Auckland region. This includes testing for GAS from: (i) primary care, and (ii) school-based sore throat management programs that are part of the RFPP in Auckland. Between 1st April and 25th June 2015, GAS isolates were collected from throat swabs taken from children aged 5 to 15 years presenting with presumptive pharyngitis in school-based programs, as part of the previously described RFPP [6]. Children enrolled in the sore throat management programmes are at highest risk of developing ARF so tend to be of Māori or Pacific ethnicity and living in areas of high social deprivation. During the same time period, GAS skin isolates were collected from children aged 5 to 15 years who presented to a general practitioner with a presumptive skin infection. These samples were collected from primary care practitioners from the same geographic region (i.e. areas of high social deprivation) as the throat swabs.

In order to provide a comparator population, GAS isolates were collected between 1st April and 30th May 2015 from throat swabs taken from children aged 5 to 15 years in Dunedin, a city in the South Island of New Zealand. These GAS isolates were collected from children presenting to a general practitioner with presumptive pharyngitis. The population in Dunedin is predominantly European (84 %), followed by Māori (7 %), Asian (6 %) and Pacific Peoples (2 %) [12]. The incidence of ARF in children in Dunedin is negligible, with only one case of ARF reported in the last five years.

Basic demographic data were collected about each child who had GAS isolated, including age, sex, ethnicity and socioeconomic status, as determined by the New Zealand deprivation index (NZDep). The NZDep score is an area-based measure of socioeconomic deprivation derived from New Zealand census data, which assigns a decile ranking from 1 to 10, with 10 representing the most deprived neighbourhoods [13].

### Bacterial isolates and molecular typing

Throat and skin swabs were plated onto tryptic soy sheep blood agar and incubated in 5 % CO2 overnight at 37 °C. GAS isolates were identified using a MALDI-TOF MS Biotyper (Bruker, Germany) and forwarded to the Invasive Pathogens Laboratory at the Institute of Environmental Science and Research, Wellington, New Zealand for further analysis. Polymerase chain reaction (PCR) and DNA sequencing of the *emm* gene was performed using previously described methods [3]. *Emm* clusters, along with *emm* patterns, were extrapolated from *emm* typing results [4].

### Statistical analysis

Simpson’s index of diversity was used to assess variation in *emm* types. This index indicates the probability that two *emm* types randomly selected are of different types–i.e. the higher the index, the greater the diversity of *emm* types in a particular population [14]. 95 % confidence intervals (CI) for the Simpson’s index were calculated as previously described [15]. Statistical analysis was performed using GraphPad Prism (Version 6), and a *p* value of 0.05 was considered significant.

### Ethical approval

As this study involved de-identified patient data, ethical approval was not deemed necessary by the Health and Disability Ethics Committees, New Zealand.

## Results

### Population demographics

A total of 452 isolates (246 pharyngeal isolates from Auckland, 104 skin isolates from Auckland and 103 pharyngeal isolates from Dunedin) were collected from 452 children. In Auckland, over 80 % of children in both groups (i.e. those with either a pharyngeal or skin isolate) were either Māori or Pacific, and predominantly resided in areas of high socioeconomic deprivation placing them in the high-risk group for developing ARF (Table 1). In contrast, over 80 % of children with a pharyngeal isolate in Dunedin were European, and resided in areas of low and moderate socioeconomic deprivation placing them in the low-risk group for developing ARF (Table 1).

**Table 1:** Demographic characteristics of children with group A Streptococcal pharyngeal and skin isolates

Characteristic	Pharyngeal isolates, Auckland (*n* = 246) (%)	Skin isolates, Auckland (*n* = 104) (%)	Pharyngeal isolates, Dunedin (*n* = 103) (%)
Age, median, years [IQR]	8.4 [6.4–11.1]	7.4 [6.1–10.0]	8.2 [7.2–9.2]
Male sex	125 (51)	52 (50)	51 (51)
Ethnicity			
European / Other	27 (11)	11 (11)	85 (82)
Māori	68 (28)	41 (39)	13 (13)
Pacific	147 (60)	47 (45)	0
Asian	4 (1)	5 (5)	5 (5)
NZDep			
Low	3 (1)	6 (6)	33 (32)
Medium	14 (6)	25 (24)	44 (43)
High	229 (93)	73 (70)	26 (25)

### *emm* typing and cluster analysis

Amongst the 452 isolates, a total of 83 *emm* types were detected, belonging to 20 *emm* clusters (Additional file 1: Table S1). Amongst Auckland isolates, the Simpson’s index did not differ significantly (on the basis of non-overlapping confidence intervals) between pharyngeal isolates (96.3 % [95 % CI 95.5 to 97.1 %]) and skin isolates (96.3 % [95 % CI 95.3 to 97.3 %]). However, the Simpson’s index was significantly lower in pharyngeal isolates from Dunedin (87.5 % [95 % CI 86.1 to 88.9 %]).

**Fig. 1:**
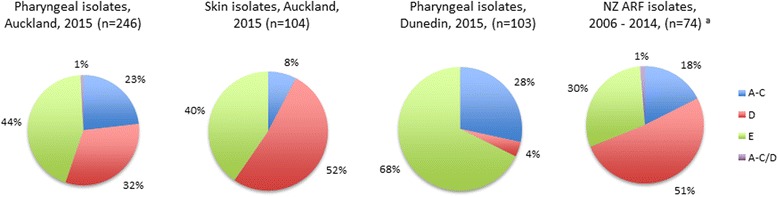
*emm* patterns of group A streptococcal strains associated with pharyngitis, skin infections and rheumatic fever in New Zealand. The rheumatic fever isolates (*) were published previously [7]

There were notable differences in *emm* cluster distribution according to specimen site and geographical location, with cluster D4 more common amongst skin isolates (36 % in Auckland skin isolates vs. 15 % in Auckland pharyngeal isolates vs. 3 % in Dunedin pharyngeal isolates), and clusters A-C4 and E4 more common amongst pharyngeal isolates, particularly from Dunedin (Table 2). Strains belonging to pattern D (skin pattern) made up 52 % of isolates recovered from skin, 32 % of pharyngeal isolates in Auckland, 4 % of pharyngeal isolates from Dunedin and 44 % of ARF associated strains reported in a previous study in New Zealand [7] (Fig. 1).

**Table 2:** *emm* clusters of group A Streptococcal strains isolated from children in New Zealand, 2015

Cluster	Pharyngeal isolates, Auckland (*n* = 246) (%)	Skin isolates, Auckland (*n* = 104) (%)	Pharyngeal isolates, Dunedin (*n* = 103) (%)	*P* ^a^
A-C2	-	1 (1)	-	NS
A-C3	24 (10)	4 (4)	7 (7)	NS
A-C4	30 (12)	1 (1)	21 (21)	<0.01
A-C5	1 (<1)	-	1 (<1)	NS
D1	6 (2)	-	-	NS
D2	9 (4)	5 (5)	-	NS
D3	1 (<1)	-	-	NS
D4	38 (15)	37 (36)	3 (3)	<0.01
E1	4 (10)	1 (1)	6 (6)	NS
E2	13 (5)	9 (9)	3 (3)	NS
E3	56 (23)	18 (17)	4 (4)	<0.01
E4	24 (10)	6 (6)	51 (50)	<0.01
E5	1 (<1)	-	-	NS
E6	27 (11)	19 (18)	7 (7)	0.018
M105	2 (<1)	-	-	NS
M111	3 (1)	-	-	NS
M19	1 (<1)	2 (2)	-	NS
M218	2 (<1)	-	-	NS
M233	1 (<1)	-	-	NS
M74	3 (1)	1 (1)	-	NS

^a^ Significance values determined by 3 × 2 *χ* ^2^ test

NB–*emm* clusters are based on those proposed by Sanderson-Smith et al. [4]

### Theoretical M-protein vaccine coverage

The theoretical coverage of the experimental 30-valent M-protein vaccine was lower for skin-associated isolates than for pharyngeal isolates (Table 3). Amongst Auckland isolates, theoretical coverage increased significantly in both groups when the potential effect of cross-opsonic antibodies was considered, although still only covered approximately half of skin isolates. There was a significant difference in theoretical vaccine coverage between pharyngeal isolates from Auckland and those from Dunedin, with coverage almost twice as high amongst GAS isolates from Dunedin compared to Auckland (Table 3).

**Table 3:** Theoretical coverage of 30-valent vaccine in relation to Group A *Streptococcus*-associated clinical syndromes

	Pharyngeal GAS isolates, Auckland; *n* = 246 (95 % CI)	Skin GAS isolates, Auckland; *n* = 104 (95 % CI)	Pharyngeal GAS isolates, Dunedin; *n* = 103 (95 % CI)
Theoretical 30-valent coverage	48.4 % (42.2 %–54.6 %)	33.7 % (25.3 %–43.2 %)	93.2 % (86.4 %–96.9 %)
Theoretical additional coverage with cross-opsonic effect ^a^	69.5 % (63.4 %–74.9 %)	54.8 % (45.2 %–64.0 %)	95.1 % (88.9 %–98.2)
Proportion of isolates belonging to *emm* types not tested for cross-opsonic effect ^a^	27.6 % (22.4 %–33.6 %)	38.5 % (29.7 %–48.1 %)	2.9 % (0.6 %–8.6 %)

^a^ As determined from references [8, 9] in which a percentage killing of 50 % or greater is considered significant in bactericidal assays

## Discussion

This study describes the contemporary molecular epidemiology of GAS strains circulating amongst high-risk children in a setting with a high burden of ARF, compared with strains amongst children residing in the same country, with minimal risk of ARF [2]. As expected, *emm* pattern D, classically associated with skin tropism, predominated in GAS skin isolates in high-risk children accounting for approximately half of all *emm* types. Somewhat surprisingly, approximately one-third of strains from high-risk children in Auckland with presumptive GAS pharyngitis also belonged to *emm* pattern D. This is in marked contrast to the distribution observed in pharyngeal isolates from low-risk children in Dunedin, where pattern D strains comprised only 4 % of isolates. Though *emm* pattern type has been described as an imperfect marker for tissue tropism [5], the high prevalence of *emm* pattern D strains observed in high-risk children in this study resembles observations in Aboriginal children in Australia, another Indigenous population with high ARF risk. A study in three remote Aboriginal communities found *emm* pattern D strains accounted for 53 % of pyoderma isolates and 24 % of pharyngeal isolates [16].

Clear differences were also observed in *emm* cluster types between high-risk and low risk children in this study. The *emm* clusters observed in pharyngeal isolates from low-risk Dunedin children closely resemble findings from other developed countries. [17]. For example, Shulman et al., recently applied the *emm* cluster system to pharyngitis isolates collected between 2000–2007 in the United States and found that clusters E4, A-C3 and A-C4 comprised 62.5 % of pharyngeal isolates, with isolates from cluster D4 representing <1 % of isolates [17]. In our study, clusters E4, A-C3 and A-C4 comprised 79 % of pharyngeal isolates from Dunedin, but only 45 % of pharyngeal isolates from Auckland. Isolates from cluster D4 accounted for just 3 % of pharyngeal strains in low-risk Dunedin children in our study but 15 % of pharyngeal strains in high-risk Auckland children and were the most prevalent cluster amongst GAS skin isolates comprising 36 % of strains. The *emm-*cluster D4 comprises 32 *emm-*types [4], and was also the most prevalent cluster in a recent analysis of GAS isolates collected in Fiji where it accounted for over 30 % of strains [18].

The high proportion of *emm* pattern D and cluster D4 amongst GAS skin and pharyngeal isolates in Auckland (an area with the highest rates of ARF in New Zealand) is in keeping with recent work describing a high proportion of *emm* pattern D and cluster D4 isolates amongst ARF-associated GAS strains in New Zealand [7]. Taken together these observations add further weight to the hypothesis that skin infections may play an important etiological role in the pathogenesis of ARF [7, 19]. Although the biological pathway is unclear, it is possible that colonizing GAS skin isolates may passage into the pharynx, or alternatively antecedent skin infection may ‘prime’ the immune system in an, as yet, uncharacterized manner, and contribute to the autoimmune process characteristic of ARF [20].

Previous studies have shown the theoretical coverage of the 30-valent GAS vaccine is reduced in low-income countries where the diversity of *emm*-types is highest [21]. In this study, vaccine coverage has been compared between different populations within the same country. The theoretical coverage was low amongst isolates from high-risk Auckland children for which the Simpson’s index for strain diversity is high, although did increase when the putative effect of cross-opsonic antibodies was considered. However approximately 40 % of skin-associated isolates and 28 % of Auckland pharyngeal isolates have not yet been tested for potential cross-opsonic activity [10, 11]. In contrast, the theoretical vaccine coverage for pharyngeal isolates from low-risk children in Dunedin is 93.2 % before taking into account the added potential effect of in vitro cross-opsonization. The high theoretical coverage in this low-risk population likely reflects the similarities in *emm-*clusters observed with pharyngeal strains from the US [17], as the 30-valent vaccine was designed to include *emm-*types commonly associated with pharyngitis in North America [10]. These findings demonstrate the importance of broad population sampling when assessing theoretical vaccine coverage.

There were a number of limitations with this study. It was assumed that GAS pharyngeal isolates were from children with pharyngitis, although information on clinical symptoms was not available for each child. Within the school-based programme, children are encouraged to present for assessment when they have a sore throat, though it remains possible that some of these isolates represent GAS colonization with concurrent viral pharyngitis, rather than true, serologically confirmed, GAS pharyngitis [22]. Similarly, clinical information about each child who had a skin swab submitted for testing was not available. However, as these swabs were submitted as part of a primary care consultation, it is likely that the majority of children had clinical symptoms suggestive of a skin infection. Moreover, our sampling period was over a relatively short timeframe; previous work, including a study from New Zealand, has demonstrated temporal variation in the proportional distribution of GAS *emm* types.

## Conclusions

In summary, this study describes a high diversity of *emm* types and a high proportion of *emm-*pattern D and cluster D4 isolates amongst children at high risk of ARF in New Zealand. These findings have potential impact on the theoretical coverage of multivalent M-protein vaccines in high-risk populations in New Zealand. In contrast, pharyngeal isolates from children at low risk of ARF within the same country were less diverse, less likely to be *emm* pattern D, and more likely to be theoretically covered by the 30-valent M protein vaccine. The high proportion of *emm* pattern D GAS strains amongst skin and pharyngeal isolates from children at high risk of ARF further highlights the potential role of skin infections in the pathogenesis of ARF.

## Additional file

Additional file 1: Table S1. Group A streptococcal isolates and *emm* types included in this study. (XLSX 12 kb)
